# Determination of Spectral Characteristics and Moisture Distribution in Wheat Grains After Sorption, Thermal, and Natural Drying

**DOI:** 10.3390/ijms26188952

**Published:** 2025-09-14

**Authors:** Timur Yu. Ivanenko, Elena V. Fomenko, Evgeny V. Morozov, Aleksander N. Matsulev, Maxim A. Lutoshkin, Nicolay P. Shestakov, Vasiliy F. Shabanov

**Affiliations:** 1 Institute of Chemistry and Chemical Technology, Federal Research Center, Krasnoyarsk Science Center of the Siberian Branch of the Russian Academy of Sciences, Akademgorodok 50/24, 660036 Krasnoyarsk, Russia; timivonk@gmail.com (T.Y.I.); morozov_if@mail.ru (E.V.M.); matsulev@mail.ru (A.N.M.); maximsfu@yahoo.com (M.A.L.); 2 Federal Research Center, Krasnoyarsk Science Center of the Siberian Branch of the Russian Academy of Sciences, Akademgorodok 50, 660036 Krasnoyarsk, Russia; shabanov@ksc.krasn.ru; 3 L.V. Kirensky Institute of Physics, Federal Research Center, Krasnoyarsk Science Center of the Siberian Branch of the Russian Academy of Sciences, Akademgorodok 50/38, 660036 Krasnoyarsk, Russia; nico@iph.krasn.ru

**Keywords:** wheat grains, seed, moisture content, sorption drying, MRI

## Abstract

The seed drying process is one of the most important aspects of post-harvest treatment, which determines the quality of the final product, cost accounting, and storage capacity. Sorption drying is of great scientific and practical importance due to its ability to gently remove moisture, which improves seed quality and ensures energy efficiency. In this study, wheat grains with an initial moisture content of 22% were dried to a moisture content of 13% using sorption, thermal, and natural air drying. The seed germination capacity after drying was 97%, 93%, and 95%, respectively. The effect of different drying methods on the morphological characteristics, microstructure, and moisture content of wheat grains was studied using a combination of experimental techniques. ATR-MIR and MAS NMR analysis revealed the biochemical stability of sorption-dried grains and the complete preservation of characteristic protein amide bands, indicating the absence of molecular degradation. Statistically significant differences in wheat grains after thermal and sorption drying were observed in luminescence peak intensities and standard deviation of the main spectral band’s half width. The MRI method demonstrated that sorption drying preserves optimal grain tissue microstructure while maintaining proper moisture levels and distribution prior to germination, as well as supporting natural mass transfer processes and moisture distribution evolution during dehydration.

## 1. Introduction

Wheat grains constitute a critical component of food security and sustainable agricultural development. Their quality is directly determined by post-harvest processing conditions, particularly drying parameters [1,2]. Research on grain drying methods is of paramount importance, as the selected regime governs key agronomic traits: germination vigor, laboratory germination capacity, and long-term seed viability [3,4].

The drying of seeds tends to be carried out in more gentle conditions compared to the drying of food grains. Significant disadvantages of traditional thermal drying are high energy consumption and loss of seed germination [5,6,7]. In particular, convection drying of grain using industrial hot air systems accounts for about 60% of total energy consumption, which significantly exceeds the average energy consumption for tillage (16%), planting and cultivation (12%), harvesting (6%), and transportation (6%) [8]. Furthermore, it has been well established that heat drying affects the physiological properties of seeds [3,5,6,7]. Managing overheating during this process can be difficult and may result in significant losses of seed quality [3,4,9,10].

In addition to traditional convective (thermal) drying methods widely used in agriculture, modern agro-industrial practices also include natural (atmospheric), vacuum, infrared, freeze-drying, as well as an innovative contact drying techniques using sorbents as desiccants [5,6,7,9,10,11,12]. The latter approach holds significant scientific and practical relevance due to its capacity for gentle moisture removal without thermal exposure. The mechanism involves direct seed contact with hygroscopic materials, thereby eliminating risks of thermal degradation in bioactive compounds. This technology ensures uniform dehydration throughout the seed volume, preservation of structural tissue integrity, and precise control of final moisture content compliant with long-term storage requirements [9,11,12].

Optimization of contact drying processes for wheat using advanced sorbent materials not only enhances grain quality preservation but also significantly reduces energy consumption in post-harvest processing compared to conventional thermal methods [8,10,13]. Research in this field is gaining particular relevance amid climate change and the growing demand for resource-efficient technologies. Consequently, the development of contact sorption-drying methods for wheat grains has emerged as a key area of scientific and technological advancement in agriculture and the food industry [9,14,15].

Wheat grains exhibit a complex structure comprising three principal components: the embryo, starchy endosperm, and aleurone layer. This structural organization renders them particularly susceptible to conventional thermal drying methods [9,16,17]. The application of contact sorption-drying technology provides the following: optimal preservation of embryonic viability; maintenance of enzymatic activity in the aleurone layer; and retention of endosperm nutritional value. Special consideration in technology development requires accounting for biochemical specificities: high starch concentration in the endosperm; substantial lipid content in the embryonic tissue; and necessitating meticulous sorbent selection and dehydration parameter optimization.

The efficiency of contact sorption drying directly depends on the physicochemical properties of the desiccant used. Magnesium sulfate is one of the best desiccants; its advantages include neutrality, high water sorption rate, high water capacity, and low regeneration temperature [18,19]. Previous studies have considered sorption drying of wheat seeds using one of the most stable natural forms of magnesium sulfate, granular kieserite MgSO_4_·H_2_O [11]. Due to its chemical composition, kieserite does not need pre-regeneration before the drying process. Its granular shape also prevents seeds from sticking to the surface, making separation after drying easier using a standard sieving method. Kieserite has been shown to retain stable sorption capacity during multiple drying cycles and even after regeneration.

Under laboratory conditions, wheat with an initial moisture content of 22 wt % was dried in contact with kieserite using an automatic mixing unit until a final moisture content of 14 wt % was achieved, which is essential for safe seed storage. After sorption drying, the germination capacity was 97% [11]. This value is typical for high-quality seeds and is higher than that obtained from seeds that have been air-dried. Based on these findings, it was concluded that contact with kieserite does not adversely affect wheat seed germination, and that sorption drying provides a non-thermal, efficient method for drying various agricultural crops. These results served as the foundation for further pilot testing of wheat contact drying during harvest.

To solve the complex problem of improving the quality and preservation of seeds, it is necessary to scientifically justify the impact of dehydration conditions on grain microstructure during sorption drying compared with traditional convection drying under heating. Wheat grains that have undergone natural air drying, where the effect on the seed microstructure is minimal, should be used as control samples.

Modern physicochemical analytical methods play a pivotal role in seed research by enabling a non-destructive assessment of biochemical composition, morphological characteristics, and physiological properties. Infrared spectroscopy (IR spectroscopy) serves as a highly efficient rapid method that provides sensitive determination of seed chemical composition and quantitative content of individual components [20]. FTIR spectroscopy is recognized as a simple, rapid, cost-effective, and non-destructive analytical tool [21].

Fluorescence spectroscopy stands out as one of the most effective techniques for evaluating and comparing structural characteristics of seed surfaces [22]. As the seed coat constitutes a cellulosic polymer, the moisture content in the surface boundary layer significantly affects spectral properties by modulating the intensity of principal bands in luminescence and excitation spectra, inducing band broadening due to increased degrees of freedom in electron-vibrational levels resulting from hydration shell formation. These effects enable precise assessment of seed surface hydration.

Another technique which effectively complements the spectroscopic approaches is Nuclear Magnetic Resonance (NMR). By detecting the magnetic properties of atomic nuclei, NMR provides detailed insights into the chemical environment of compounds such as carbohydrates, proteins, lipids, and water within seeds [13]. In addition, Magic-Angle Spinning (MAS NMR) is a specialized technique that enhances resolution in solid-state NMR by spinning the sample at the “magic angle” (54.74° relative to the magnetic field). This method reduces line broadening caused by anisotropic interactions, allowing for clearer analysis of rigid or semi-solid components in wheat seeds, such as starch granules or cell wall polymers.

Another modern experimental technique based on NMR is Magnetic Resonance Imaging (MRI). This method has proved to be very informative means for the non-destructive study of optically opaque samples due to accurate visualization of their internal structure and morphology which was widely accepted in medical diagnostics [23]. Apart from medical applications, the MRI method was found very effective for studying various materials and processes including (but not limited to) polymers and composites, porous media, colloids, multiphase multicomponent systems, as well as processes of heat and mass transfer, swelling, drying, gelation, etc. [24,25,26,27,28]. It is worth noticing that, among numerous applications, the MRI method is highly promising for studying the content, physical state, and dynamics of water in food and agricultural products, including the processes of seed moistening, swelling, and drying [13,29]. Thus, using MRI, the morphology and internal structure of seeds, state and amount of water, moisture distribution and migration, quantitative parameters, and qualitative picture of the processes in them can be visualized [13,29,30,31,32]. The high information content of the method, combined with the ability to carry out measurements in situ, allows obtaining unique, previously inaccessible data on the objects under study.

The aim of this study is to perform a comparative analysis of how different drying methods affect the morphological characteristics and microstructure of wheat grain tissues using a set of complementary physicochemical techniques. The key objectives include identifying principal differences in wheat grains spectral characteristics; performing a comparative analysis of moisture distribution in grain tissues; and establishing whether there are structural modifications in wheat grain after sorption drying compared to thermal and natural air drying.

## 2. Results

Comparative studies of morphological features and microstructure of grains were carried out on wheat dried by various methods. Sorption drying was carried out using granular kieserite (MgSO_4_·H_2_O) as a solid desiccant. For comparison, wheat grains that were traditionally processed in industrial hot air systems were used as a sample after thermal drying. Wheat grains brought to requested moisture content in an air stream without heat treatment were taken as samples after natural drying. To obtain a low-quality wheat grain, the sample was subjected to high-temperature treatment at 80 °C for 72 h. Grain drying conditions, moisture content, and seed germination are presented in Section 4.1.

### 2.1. Infrared Spectroscopy

**Figure 1:**
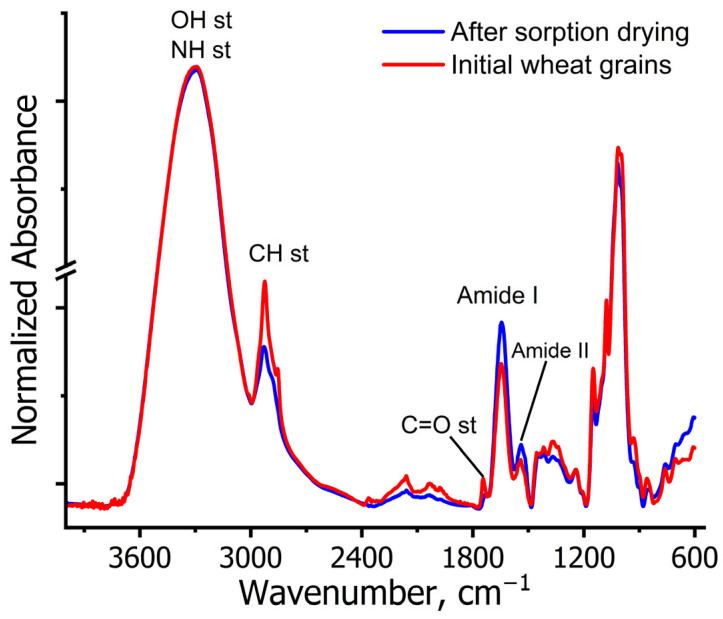
ATR-MIR spectra of wheat grains before and after sorption drying.

Attenuated total reflectance mid-infrared (ATR-MIR) spectroscopic studies were performed on the initial mature wheat grains and after sorption drying. Due to the complex chemical composition of grains, the ATR-MIR spectra reveal vibrations of various functional groups (Figure 1).

A broad band at 3500–3300 cm^−1^ corresponding to OH and NH stretching vibrations from carbohydrates and proteins [33]. A barely discernible band at 3010 cm^−1^ attributed to =CH olefinic stretching. Characteristic bands in the 2850–3000 cm^−1^ region representing symmetric and asymmetric CH_3_ and CH_2_ stretching vibrations of lipids, with contributions from proteins, carbohydrates, and nucleic acids. A distinct band at 1750 cm^−1^ assigned to C=O ester stretching vibrations of lipids [34]. Amide I band (1640 cm^−1^) reflecting β-sheet and α-helix protein conformations. Amide II band (1540 cm^−1^) arising from N-H bending vibrations. Weak bands in the 1480–1200 cm^−1^ region corresponding to C-H bending and O-H deformation vibrations of polysaccharides in the seed coat [34,35,36].

The fingerprint region (1200–600 cm^−1^) contains characteristic carbohydrate vibrations, predominantly from starch and cellulose [35]. A strong band at 1007 cm^−1^ assigned to pectin vibrations [35,36]. A weak broad feature in the 2400–1800 cm^−1^ region, potentially representing overtones or combination bands.

The ATR-MIR spectra (525–4000 cm^−1^) showed no significant changes in major absorption bands, suggesting no detectable alterations in the primary biochemical composition (proteins, lipids, carbohydrates) of sorption-dried grains. However, complementary techniques would be required to conclusively rule out molecular degradation and enable more sensitive assessment of protein stability.

### 2.2. MAS NMR Spectroscopy

**Figure 2:**
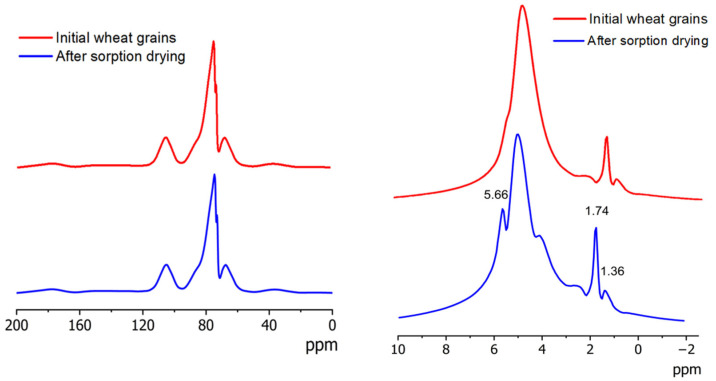
MAS NMR spectra of wheat grains before and after sorption drying (CP-MAS ^13^C (**left**), ^1^H (**right**)).

To corroborate the findings from ATR-MIR spectroscopy, solid-state NMR measurements were performed on pre-ground samples (Figure 2). The ^1^H NMR spectra of both samples exhibited well-resolved lipid signals (1.36, 1.73, 2.20, and 5.66 ppm), corresponding to the most mobile fraction of the flour [37]. As the moisture content decreased during drying, the broad water signal at 4.7 ppm diminished, making the lipid signal at 5.66 ppm and the protein signal at 4.30 ppm more pronounced in the desiccated sample [37]. The ^13^C spectrum was dominated by starch-derived signals (60–110 ppm), with very weak contributions from aliphatic (20–40 ppm) and aromatic carbons (~130 ppm). The carbonyl carbons of the peptide backbone in gluten proteins were represented by a signal at 170 ppm. However, possible contributions from protein backbone carbons to the 40–70 ppm region cannot be ruled out [37,38,39].

Overall, the spectral profiles remained largely unchanged, except for the reduced water signal intensity, supporting the conclusion that no molecular degradation of proteins, lipids, or starch occurred during the sorption drying of wheat seeds.

### 2.3. Photoluminescence Spectroscopy

**Figure 3:**
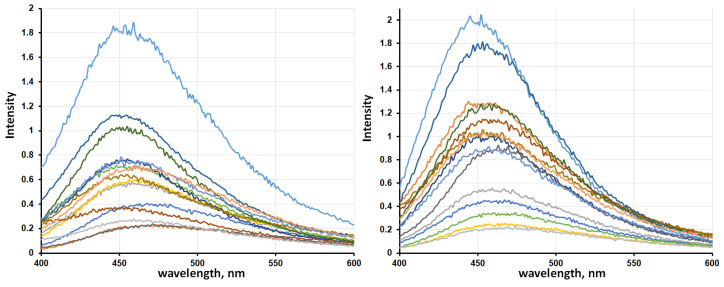
The luminescence spectra of wheat grains after sorption (**left**) and thermal (**right**) drying.

Spectrofluorimetric analysis is a highly effective method for the investigation of the structure and spectral parameters of the seed surfaces. The seed surface is the cellulose-based polymer matrix, exhibiting the highest susceptibility to structural deformations under thermal treatment. Measurements were carried out on wheat grains after traditional thermal drying using industrial hot air systems and after-sorption drying using kieserite. The grain surface after natural drying was not studied in detail using photoluminescence, as it was similar to the grain structure after industrial hot air drying. The fifteen wheat grains (N) for each drying method (sorption and thermal) were estimated, all spectra were recorded under identical conditions. To exclude interference from the embryonic region and potential grain coat defects, measurements were taken from the lateral grain surfaces. The obtained surface luminescence spectra are presented in Figure 3.

For statistical analysis, the following key spectral parameters were selected: wavelength of maximum intensity (λ^max^), peak intensity value at λ^max^, full width at half maximum (FWHM). Table 1 summarizes the quantitative spectral parameters and their statistical metrics for grains after thermal and sorption drying.

**Figure 4:**
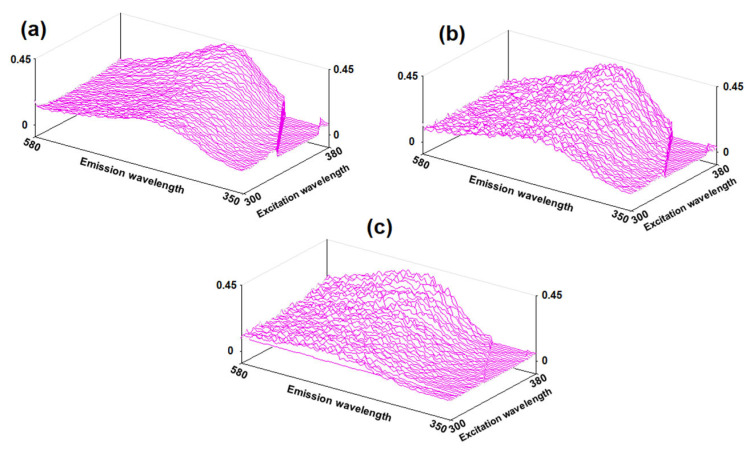
Scanned fluorescence spectra of the seed surface: (**a**)—after sorption drying; (**b**)—after thermal drying; (**c**)—after high-temperature treatment at 80 °C.

The scanned fluorescence spectra of the seed surface are shown in Figure 4. Seeds after high-temperature treatment were selected for the comparison. From Figure 4, it is evident that the seeds with minimal germination, which were treated at 80 °C, differ from others by a distracted and fragmented structure with significantly more dispersion. This indicates that the process of water removal during using a sorbent is smoother and more isotropic both in time and on the surface of the seeds (Figure 4a). Both spectra of seeds after exposure to heat (samples after thermal drying and after high-temperature treatment at 80 °C) have a greater asymmetry towards red wavelengths (Figure 4b,c). Such heterogeneity in the spectra indicates that some of the cellulose molecules on the surface of the shell have hydrated shell structures that differ significantly from the median values. This indicates that without the use of a sorbent, a noticeable moisture gradient is formed not only on the surface of the shell, but also in its adjacent layer, which also complicates subsequent embryo evaluation.

### 2.4. MRI Visualization

#### 2.4.1. Moisture Visualization of Wet Grains

**Figure 5:**
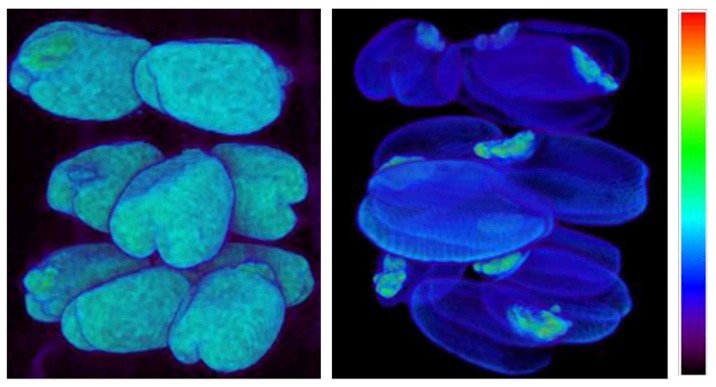
Reconstructed MRI images of wheat grains subjected to different drying technologies: surface rendering (**left**); deep contrast with clear embryo visualization (**right**). Moisturized grains in upper layer—after sorption drying, middle layer—after high-temperature drying, bottom layer—after natural air drying. The higher the signal in the images, the higher moisture content is.

In order to visualize the spatial distribution of moisture in wheat grains earlier subjected to different drying technologies and then moisturized back, 3D MRI images were acquired with subsequent program reconstruction to provide the surface rendering and deep contrast pictures (Figure 5). To this aim, grains from different batches were stacked with the grain fold (crease) downward in three layers; each layer was separated from the next one by a layer of double-sided adhesive tape which has no signal on the images. The first layer from the bottom consisted of four naturally dried grains which were laid out in the shape of figure “Ш”. The middle and top layers each consisted of three grains which were laid out in the shape of figure “Π”. Here, in the middle layer, the grains were subjected to high-temperature treatment at 80 °C, and in the upper layer, they were subjected to sorption drying with kieserite. Before obtaining MRI images, the grains were subjected to moisturizing: they were kept in one layer in an atmosphere with relative air humidity of 100% for three days at room temperature. This allowed us to achieve high moisture content of the grains without losing their quality, since longer moisturizing times mean that pathogenic fungi begins to grow on the grains’ surfaces.

The images obtained (Figure 5) demonstrated a virtually identical distribution of signal intensity among grains subjected to different drying technologies: the most intense signal, due to the maximum moisture content, is observed in the embryo region. However, it should be noted that among the grains subjected to 80 °C temperature drying, one grain was found with the absence of pronounced increased moisture in the embryo area (middle layer, foreground), which may indicate its death during high-temperature processing.

**Figure 6:**
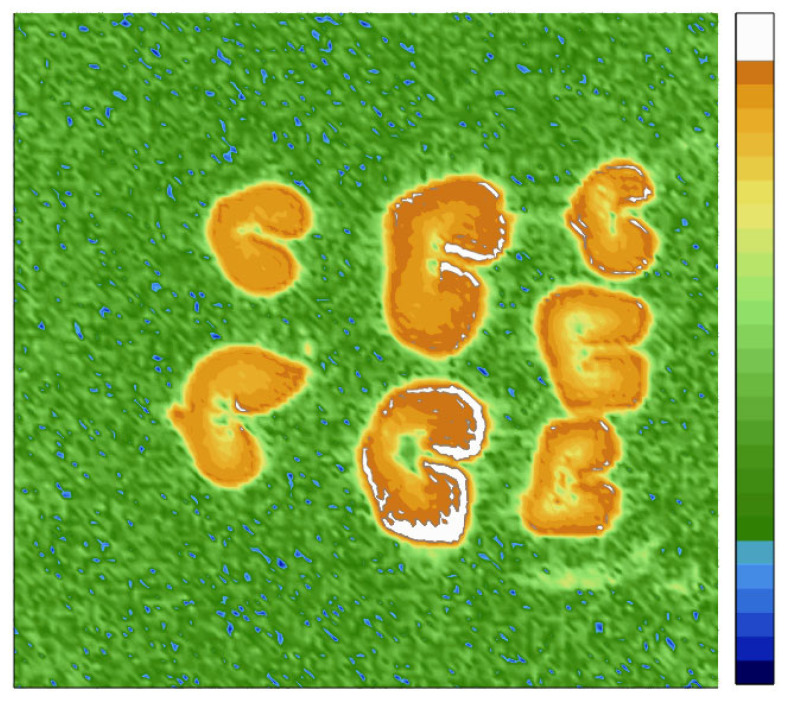
Distribution of the logarithm of signal intensity within the slices acquired across the long axis of the grain. From left to the right: columns of 2 slices of grains after sorption and high-temperature drying, respectively; a column of 3 slices of air-dried grains. The higher the signal in the image, the higher moisture content is.

Despite the absence of a qualitative difference in the degree of moisture content of grains subjected to different drying technologies, a more detailed analysis showed some differences in the quantitative distribution of signal intensity. It was found that for the same moisturizing time, the average visual signal intensity of grains subjected to high-temperature drying reached the highest value (Figure 6). The average signal intensity (M) calculated over the grain cross-section area is shown on slices in Figure 6, demonstrating that grains after sorption drying, high-temperature drying, and natural air drying have M = 6.6 ± 0.2, 8.2 ± 0.3, 6.8 ± 0.3, respectively. This data corroborates the visual differences observed. It is necessary to note that apparent difference in the size of the grains seen in Figure 6 comes from the fact that MRI slice comes through non-equivalent sections of the grains stacked into three layers. One could see in Figure 5 that all grains have a similar size.

The results in Figure 6 also demonstrate the presence of the moisture gradient: the signal intensity decreases from the highest values at the outer grain boundaries to the lowest values located in the inner part of the grain. In this case, the largest gradient is visualized in grains subjected to high-temperature drying. It can be seen that exposure to high-temperatures changes both the overall moisture content of the grain and the moisture distribution, which is most probably due to changes in the grain tissue microstructure under the action of high temperatures. Unlike high-temperature drying, the treatment of the grains with an adsorbent has little effect on the average moisture content as well as the distribution of moisture throughout the grains structure, making such treatment close to the conditions of natural air drying.

#### 2.4.2. Moisture Visualization During Grains Drying

As is shown in the previous subsection, the sorption-drying technology facilitates preserving the grain microstructure, which makes such processing close to the conditions of natural air drying. In order to establish the degree of identity of the processes occurring during grain drying under natural conditions and during sorption drying, MRI studies of the moisture distribution in grain and the dynamics of its change over time were carried out. To this aim, 2D MRI images were acquired during drying processes in situ: two batches of moisturized wheat grains (22 wt % of moisture content) were placed into MRI probe and consequently the natural air drying (30 °C, without forced air flow, the first batch) and then sorption drying (22 °C, granulated kieserite, the second batch) were performed with periodic acquisition of MRI images.

**Figure 7:**
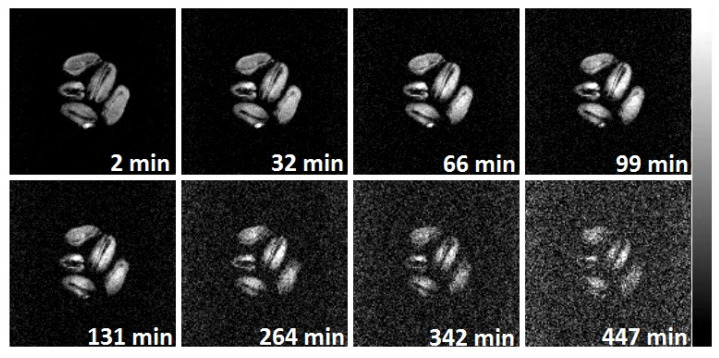
MRI images of wheat grains during natural air drying at 30 °C. The time elapsed from the beginning of the drying process is indicated. Higher signal intensity corresponds to higher moisture level.

It was found that during the grain drying process in conditions similar to those occurring for natural air drying, there is a gradual decrease in the signal intensity on the images (Figure 7). Thus, after 4 h of drying, the signal intensity decreases significantly, which is clearly visible from the increased noise level on the images; finally, after 7 h of drying at 30 °C, only small areas remain in the grain that retain the signal. Using the weighing, it was established that a complete loss of signal is observed when the absolute grain moisture reaches 8–10 wt %.

**Figure 8:**
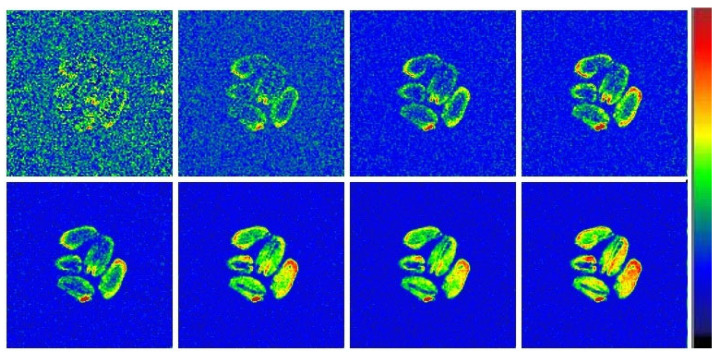
Differential images of wheat grains during air drying at 30 °C which demonstrate the accumulated changes in the signal intensity as compared to original image acquired before the drying. The order of the images is the same as is shown in Figure 7. Higher signal intensity corresponds to the bigger difference between original and current images, i.e., decreased moisture content.

For a more detailed analysis of the drying process, two types of differential images were calculated (Figure 8). In the first type, each picture presented was obtained by algebraic subtraction of the 2D MRI image acquired at a certain point in time from the original one (obtained right before the start of drying). It is clearly seen that the drying process is initiated by the grain surface and as the process proceeds, the decrease in moisture content (increase in the differential signal intensity) begins to spread into the depth of grain.

**Figure 9:**
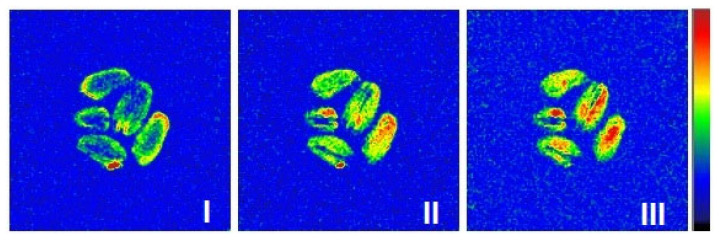
Differential images of wheat grains during air drying at 30 °C which demonstrate the changes in the signal intensity between two consecutive MRI images on various stages of the drying process. Higher signal intensity corresponds to bigger difference between two consecutive images, i.e., decreased moisture content.

The second type of differential images was obtained by algebraic subtraction of two consecutive 2D MRI images acquired one by one in time, i.e., this type captures the “instantaneous” dynamics of the processes of changing the moisture content in the grain. Using these instantaneous pictures, it was observed that the drying process actually occurs in three stages (Figure 9). At the first (I) stage, the most pronounced change in moisture content appears mainly in the vicinity to the outer boundary of the grain, which is clearly visible via the bright outline. At the second (II) stage, moisture begins to actively migrate from the internal areas of the grain to the drying shell, which is expressed in a relatively uniform change in the signal throughout the entire volume of the grain and the disappearance of the bright outline (Figure 9). Finally, at the third (III) stage of the process, the greatest changes in moisture content are found in the internal region, which is clearly seen from the highest signal intensity inside the grain.

**Figure 10:**
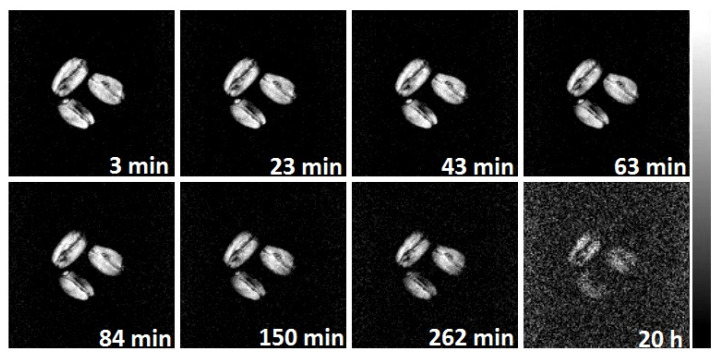
MRI images of wheat grains during sorption drying at 22 °C. The time elapsed from the beginning of the drying process is indicated. Higher signal intensity corresponds to higher moisture level.

Acquisition of MRI images of the grains during sorption drying in situ showed an overall picture similar to that described above for natural drying. Thus, a gradual decrease in signal intensity and, accordingly, grain moisture content is observed on the images (Figure 8). However, the drying process proceeds slightly slower, which is evident from the relatively low noise level after 4 h of drying. Even after 20 h of drying, there are still areas of visible signal in the grain (Figure 10).

**Figure 11:**
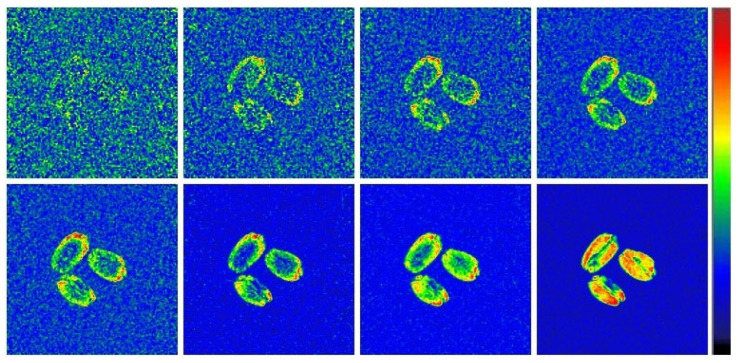
Differential images of wheat grains during sorption drying at 22 °C which demonstrate the accumulated changes in the signal intensity as compared to original image acquired before the drying. The order of the images is the same as is shown in Figure 8. Higher signal intensity corresponds to a bigger difference between original and current images, i.e., decreased moisture content.

The calculation of a series of differential images of the first type (demonstrating the accumulated changes in the signal intensity) during the sorption drying of the grains confirmed the identity of the changes in the signal distribution to those observed in the case of air drying. It should be noted that, due to the slower dynamics of moisture migration during sorption drying, the initiation of the drying process by the grain surface can be more clearly visualized (Figure 11). Moreover, over a sufficiently long time of sorption drying, the cumulative change in the signal has its maximum value exactly in the near-surface regions of the grain.

**Figure 12:**
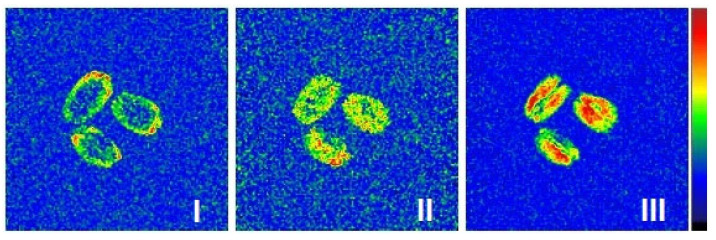
Differential images of wheat grains during sorption drying at 22 °C which demonstrate the changes in the signal intensity between two consecutive MRI images on various stages of the drying process. Higher signal intensity corresponds to a bigger difference between two consecutive images, i.e., decreased moisture content.

Visualization of the “instantaneous” dynamics of the moisture changing in grain by means of differential images showed the same three-stage process for sorption drying (Figure 12). Again, due to the slower dynamics of moisture migration during the process, individual stages are seen much more clearly. Thus, the changes in moisture content at the first stage practically does not affect the internal areas of the grain (Figure 12), and at the second stage, the changes in moisture distribution inside the grain are of very uniform nature. The second stage is then followed by an inversion of the moisture content dynamics, i.e., the central part of the grain undergoes to the most pronounced moisture loss at the third stage of the process. As a result, the use of sorption-drying technology yields the evolution of the moisture distribution within the grains to be very similar to that formed under natural air-drying conditions.

## 3. Discussion

ATR-MIR and MAS NMR analysis confirmed the biochemical stability of sorption-dried grains, with spectral patterns showing the following: (1) no band disappearance, (2) no new band formation, and (3) complete preservation of characteristic protein amide bands, collectively indicating absence of molecular degradation.

Analysis of grain surfaces after thermal and contact drying revealed that the observed fluorescence signals originate primarily from polysaccharides (mainly starch) in the grain coat composition. The mean values of luminescence spectrum maxima were nearly identical (459 nm for sorption drying and 461 nm for thermal drying), though the confidence intervals (±Δ) differed by 0.1 units. The fluorescence intensity of grain surfaces was notably higher for thermal drying (0.97 compared to 0.68 for sorption drying) with comparable confidence intervals and standard deviations. Significant differences were observed in spectral line width parameters: 110 nm for sorption drying versus 100 nm for thermal drying. The increased line width typically correlates with enhanced vibrational–rotational energy levels, which in this case may be explained by higher water content on grain surfaces after sorption drying. The decreased intensity of the main absorption peak may also result from increased hydration of the surface layer: the formation of hydration shells causes the quenching of luminescence in cellulose matrix chromophores. It should be noted that the variation in main peak width values (by confidence interval and SD) was greater for sorption-dried grains, while thermal drying produced more uniform parameters. The reasons for this difference can be found in the nature of sorption drying—magnesium sulfate can extract only water molecules which are weak bonding with the polymer template. These water molecules do not have hydrogen bonds with the starch template and are linked through an electrostatic mechanism as dipole points. The energy of the formation of donor–acceptor bonds between Mg^2+^ ion and H_2_O is not enough for the destruction of the inner hydration shell of the polymer template. At the same time, during thermal drying, destruction occurs in both parts of water: H_2_O molecules from the inner hydration shell as well as weak-linked molecules.

Along with spectral characteristics, MRI studies of the spatial distribution of moisture in grains subjected to different drying technologies were carried out. It was found that, despite the qualitatively similar picture of moisture distribution in grains, the high-temperature treatment contributes to a change in the microstructure of grain tissues, which leads to their greater moisture capacity, especially in the near-surface areas which have experienced the greatest impact of the temperature. As a result, the balance of moisture distribution between the grain surface and its interior changes, which significantly distinguishes the grains after high-temperature drying from the grains dried under natural air conditions. As it was previously observed, the high temperature affects the plant tissues and grain that significantly changes their microstructure, inevitably leading to a change in such characteristics as moisture capacity, moisture absorption rate, etc. [40,41,42]. These changes are caused by the processes of polysaccharide dehydration (primarily starch) and protein denaturation, which leads to an increase in the degree of crystallinity, a change in the spatial conformation of molecules, and the destruction of fibrils and three-dimensional texture of tissues. Unlike the high-temperature drying, the treatment of grain with an adsorbent has virtually no effect on either the average moisture content of the grain or the distribution of moisture over its structure, making such treatment close to the conditions of natural air drying.

A comparison of the moisture content and its distribution in wet grains after different types of drying clearly indicates superior performance of the sorption-drying technology as compared to high-temperature one. However, to confirm the identity in the character of mass transfer processes between the natural air drying and sorption-drying technologies, MRI studies of grain drying were carried out in situ. It was shown that during sorption drying, the changes in the distribution of moisture inside the grains are very similar to those observed in the case of natural air drying. In particular, the moisture loss, which is first detected in the near-surface layers of the grain and only then spreading to entire grains volume, occurs in three stages: beginning from the moisture transport outward from the near-surface regions, it proceeds in the direction of the moisture gradient to equalize the moisture content between the near-surface regions and the central part, and ends up with the prevalence of moisture loss by the central part over the near-surface regions.

It has been established earlier that the main mechanism of moisture transport in grain is the molecular diffusion of water and vapor under the influence of the concentration gradient, vapor pressure, and capillary forces [43,44]. According to literature data, the grain drying process typically exhibits two different stages, based on changes in the drying rate: the constant rate and the falling rate stages [45,46]. At the constant rate drying period, the evaporation rate of moisture from the grain surface remains relatively constant, determined by the diffusion of water vapor; at this point, the internal moisture distribution remains stable, and there is a significant moisture gradient. At the falling rate period, the surface moisture gradually decreases, and internal moisture begins to migrate to the surface; this stage is controlled by the migration and evaporation of moisture from the interior to the exterior of the grain, with the moisture gradient gradually decreasing and the drying rate significantly slowing down.

Dividing the drying process into three conditional stages was made in this work for the first time; however, the MRI observation of the features of moisture loss at the last stage of the drying process was described earlier. It turned out that at the late stages, the rate of moisture migration inside the grain significantly advances the rate of its loss from the surface [47]. Obviously, the observed changes in signal intensity, caused by changes in local grain moisture, are in good agreement with the diffusion nature of drying processes under the action of moisture gradient. The only difference is that in natural air drying, the moisture gradient arises as a result of free evaporation of water from the grain surface, while in sorption-drying technology, the gradient arises due to the loss of water molecules which become captured by the adsorbent on the grain surface.

It is necessary to note that the qualitative nature of the results obtained is an intrinsic feature of the MRI method. MRI deals with images which demonstrate the internal structure of the optically opaque samples, showing up the apparent distribution of the NMR signal determined by the morphology of tissues and compartments. Meanwhile, extracting some quantitative data from MRI images is not always possible. For example, signal intensity obviously correlates with moisture content in grains, but the relationship is complex that attempts to calculate moisture content in situ directly from the images completely fail. The problem is that the signal intensity, M, is the product of spin–lattice, spin–spin relaxation times, and proton density from the one side, and instrumental parameters such as flip angle (FA), repetition time (TR), and echo time (TE) of pulse sequence, from the other side. For this reason, MRI of biological samples often suffers from the deficiency of quantification that requires supplementing with quantitative data from the other methods (e.g., weighing), as can be seen in numerous medical studies or MRI works dealt with wheat grains [13,47].

In conclusion, MRI demonstrates that the application of sorption-drying technology ensures the preservation of the required microstructure of grain tissues, which guarantees the necessary moisture content and distribution within the grain before its germination. Since the sorption drying provides similar mass transfer and evolution of moisture distribution to processes in natural drying, it makes this technology favorable for application in plant-scale level due to its higher throughput as compared to gentle air drying at low temperatures which is difficult to scale up. Yet, it is worth noting that the methodology used in this study can be successfully extended to visualize the processes of moisture migration during the drying of other agricultural crops under different conditions.

## 4. Materials and Methods

### 4.1. Wheat Seeds

The wheat seeds (*Triticum aestivum* L.) used in this study belong to the Novosibirskaya 16 grade variety produced in the East Siberian region (EPF “Mikhailovskoye”, FRC KSC SB RAS, Krasnoyarsk, Russian Federation) and harvested in 2023.

To determine the moisture content of grain, we used the air–heat drying method in accordance with the Interstate Standard GOST 13586.5-2015 [48] and the International Standard ISO 712-1:2024 [49]. The statistical analysis of the data was performed in accordance with GOST 13586.5-2015 [48], which determines the reproducibility of experimental data.

The kieserite granulated grade, a commercial agrochemical magnesium sulfate MgSO_4_·1.3H_2_O, was used as a solid desiccant. This product is manufactured by the South Ural Magnesium Compounds Plant (UUZMS, Orenburg Region, Kuvandyk, Russian Federation), in accordance with Technical Specifications [50].

Unlike previous laboratory studies [11], in this work, wheat grains were dried on an industrial scale. During the harvesting campaign, pilot tests of the sorption drying of wheat grains were carried out. For wheat grains with initial moisture content of 22 wt %, the required for storage moisture content of 13 wt % was achieved in 4 h of sorption drying with a desiccant/grain ratio = 1:3. In parallel, wheat grains were dried in industrial hot air systems (thermal drying) and in natural air without heat treatment on pre-cleaning machines (natural drying) until the moisture content required for seed storage was 13 wt %.

The germination of wheat seeds was assessed following a standard test according to Interstate Standard GOST 12038-84 [51] and the International Rules for Seed Testing [52]. The test was carried out in four replicates, each comprising 100 seeds. The statistical analysis of the data was performed in accordance with GOST 12038-84 [51], which ensures the reliability and reproducibility of the experimental results.

According to seed quality tests [51,52], wheat germination after sorption drying was 97%. This value is higher than the germination capacity of wheat after natural drying, which was 95%. The seed germination after thermal drying was 93%, which is lower than using the other two drying methods.

To obtain low-quality seeds, wheat grains, after being thermally dried, were subjected to additional high-temperature treatment at 80 °C for 72 h. The moisture content of the seeds was 2% by weight, and the germination capacity was only 22%.

Seed sampling was carried out in accordance with the regulations established by the International Seed Testing Association (ISO 24333:2009) [53] and the Interstate Standard GOST 13586.3-2015 [54], which specifies requirements for representative samples.

For the visualization of moisture distribution in MRI experiments, the grains after high-temperature (80 °C) treatment, sorption, and natural air drying were used in wet state. Moisturizing was achieved by keeping the grains in one layer in an atmosphere with relative air humidity of 100% for 3 days at room temperature. Additionally, two batches of moisturized wheat grains (22 wt % of moisture content) were used for visualization of moisture dynamics during air drying and sorption drying inside the MRI probe at 30 °C and 22 °C, respectively.

### 4.2. ATR-MIR

Intact wheat seeds were selected and analyzed without pre-treatment. For each group (pre- and post-sorption drying), 15 seeds were sampled, with three spectra collected at different locations per seed (64 scans per measurement), followed by spectral averaging. ATR-FTIR spectra were recorded using a Vertex 70 Fourier-transform infrared spectrometer (Bruker, Ettlingen, Germany) equipped with a MIRacle ATR accessory (PIKE Technologies, Fitchburg, WI, USA) featuring a zinc selenide (diamond/ZnSe) monocrystal prism. The instrument configuration incorporated a KBr beam splitter and an RT-DLaTGS photodetector (Bruker, Ettlingen, Germany), enabling spectral acquisition in the 525–4000 cm^−1^ range with 4 cm^−1^ resolution at ambient temperature conditions.

### 4.3. NMR Spectroscopy

Ten wheat seeds were pre-selected before and after sorption drying, which were then ground into flour using an agate mortar.

High resolution ^13^C and ^1^H MAS NMR experiments were performed at 23.0 ± 0.1 °C on Bruker AVANCE III 600 spectrometer (Bruker, Ettlingen, Germany), at 600 MHz for proton and 150 MHz for carbon-13, equipped triple-channel 3.2 mm MAS probe head. ^1^H and ^13^C 90° pulse lengths were 4.0 and 1.8 μs, respectively. ^1^H spectra were recorded with 16 scans, using a recycle delay of 5 s, at MAS frequency 7 kHz. ^13^C CP-MAS experiments were carried out using recycle delay of 5 s under decoupling conditions with contact time 500 μs, a 7 kHz MAS frequency and 1600 scans. All spectra were processed in Topspin 3.2 software.

### 4.4. Photoluminescence

After thermal and sorption drying, 15 individual wheat grains from each batch of representative seed samples were analyzed. All spectra were recorded under identical conditions. Luminescence spectra were recorded using impulse stroboscopic spectrofluorometer “Fluorat-02-Panorama” (Lumex, St. Petersburg, Russia) with an external double monochromator and 1 cm quartz cell. “FROG” unit attachment for solid-state fluorometric measurements outside the cell compartment were used. Impulse duration was set to 40 µs and pause between impulses was set to 0.5 µs. Statistical analysis was performed using the GNU Octave software version 10.1.0 package employing standard statistical modules. The wavelength of excitation was 380 nm. The “±” values represent confidence limits (*p* = 0.95) throughout the article. The studied samples of seeds have a high degree of homogeneity (see Statistical Parameters, Table 1).

### 4.5. MRI

MRI experiments were repeated several times with different batches of representative wheat grain samples and results showed similar character of the processes that occurred.

All MRI experiments were carried out using NMR spectrometer Bruker AVANCE DPX 200 (Bruker, Ettlingen, Germany) in following configuration: ultrashield superconducting cryosolenoid with a magnetic field strength of 4.7 T, which corresponds to a 200.13 MHz proton resonance frequency, water-cooled and self-shielded Bruker GREAT 3/60 gradient unit, a xyz-gradient probe PH MICRO 2.5 with values of pulsed-field gradient strength up to 1 T/m, 25 mm internal diameter birdcage coil tuned and matched to ^1^H nucleus. Image acquisition and processing was carried out using ParaVision 4.0 software supplied by the manufacturer. The reconstruction of 3D-MRI images was carried out using open MIPAV program which allows adjusting the required scale, dimensions, and color scheme of the images.

For MRI visualization of the grain drying processes, the gradient echo technique (Gradient Echo Fast Imaging) was used. The parameters of image acquisition were as follows: field of view (FOV) 2.5 cm, slice thickness of 1 mm, matrices of 128 × 128 pixels, repetition time (TR) = 100 ms, echo time (TE) = 1.8 ms, image acquisition time = 4 min 16 s. For 3D MRI, the same pulse sequence and parameters were used with FOV = 1.8 × 1.8 × 1.8 cm.

The three-dimensional visualization of the MRI images using open MIPAV program was carried out using the GPU-based Volume Renderer and then Surface Volume Renderer. In this case, the transfer function of the opacity parameter, which was linear by default, was changed so that the main part of voxels with signal intensity lower than that of the embryo voxels did not obscure the embryo region. In fact, opacity was changed so that, up to approximately the maximum of the histogram of the distribution of the signal intensities of the voxels related to the objects under study (i.e., excluding background noise), opacity was equal to zero, and then increased linearly to the default maximum. The LUT (Look-Up Table) transfer function parameters were left unchanged except that the Rainbow2 LUT color palette was selected.

MRI images obtained using scanner software and experienced no post processing are presented in shades of gray according to standard imaging representation (Figure 7 and Figure 10). In this case, the signal intensity bar is shown from the right side of the figure and varies in [0–255] grade levels, i.e., the brighter intensity, the higher the signal is. Any images obtained either via post processing (Figure 5) or algebraic operations with raw data (differential images, Figure 8, Figure 9, Figure 11 and Figure 12; or logarithmic image, Figure 6) are presented in color mode with the signal intensity bar shown from the right side of the figure.

## 5. Conclusions

In this work, a comparative study of how different drying technologies, i.e., thermal, sorption, and natural drying, affect the morphological characteristics, microstructure, and moisture content of wheat grain tissues, carried out using a set of complementary experimental techniques. First, using the MRI method, it was demonstrated that sorption-drying technology preserves optimal grain tissue microstructure while maintaining proper moisture levels and distribution prior to germination, as well as supporting natural mass transfer processes and moisture distribution evolution during dehydration. Then, ATR-MIR and NMR analysis confirmed the biochemical stability of sorption-dried grains and complete preservation of characteristic protein amide bands, indicating the absence of molecular degradation. Statistically significant differences in wheat grains after thermal and sorption drying were observed in luminescence peak intensities and standard deviation of the main spectral band’s half-width. These differences suggest the quenching of cellulose luminescence by water hydration shells and increased electron–vibrational levels due to enhanced hydration in sorption-dried wheat grains. The methodology, based on combination of different experimental techniques which was used in this study, can be successfully extended to investigate the processes of moisture migration during the drying of other agricultural crops under different conditions.

## Figures and Tables

**Table 1:** Spectral and statistical characteristics of grains after thermal and sorption drying (λ^max^—wavelength of maximum spectral intensity, I^max^—maximum intensity at processed wavelength, S—standard deviation, Δ—confidence limit) for *p* = 0.95.

Sorption Drying				Thermal Drying			
N *	λ^max^, nm	I^max^	Half Weight, nm	N *	λ^max^, nm	I^max^	Half Weight, nm
1	468	0.405	120	1	467	0.456	103
2	473	0.234	128	2	455	1.277	106
3	458	0.580	109	3	459	0.555	102
4	460	0.596	112	4	468	0.253	112
5	459	1.886	106	5	452	2.047	86
6	460	0.708	113	6	474	0.347	109
7	454	0.374	126	7	458	1.442	84
8	455	0.769	98	8	458	0.999	97
9	471	0.237	78	9	463	0.926	89
10	455	0.641	109	10	453	1.056	111
11	450	1.129	104	11	453	1.815	93
12	455	1.036	93	12	457	1.277	98
13	458	0.753	113	13	457	0.914	99
14	460	0.691	119	14	460	1.025	101
15	459	0.279	122	15	474	0.231	118
Mean	459	0.688	110	Mean	461	0.970	100
S	1.65	0.11	3.25	S	1.61	0.13	2.23
±Δ	3.53	0.23	6.95	±Δ	3.44	0.28	4.78

*—individual grain number.

## Data Availability

The data presented in this study are available on request from the corresponding author.
